# Inflammation and Thyroid Cancer: Deciphering the Role of Blood Immune Indexes

**DOI:** 10.3390/cancers17081363

**Published:** 2025-04-19

**Authors:** Salvatore Sorrenti, Gregorio Scerrino, Eleonora Lori, Fabrizio Vassallo, Stefania Saverino, Calogera Amato, Giuseppina Melfa, Pierina Richiusa, Sergio Mazzola, Antonella Lopes, Giuseppina Orlando, Giuseppa Graceffa

**Affiliations:** 1 Department of Surgery, Sapienza University of Rome, Viale del Policlinico 155, 00161 Rome, Italy; salvatore.sorrenti@uniroma1.it; 2 Unit of Endocrine Surgery, Department of Surgical Oncological and Oral Sciences, Policlinico “P. Giaccone”, University of Palermo, Via Liborio Giuffré 5, 90127 Palermo, Italy; gregorio.scerrino@tiscali.it; 3 Unit of General and Emergency Surgery, Department of Surgical Oncological and Oral Sciences, Policlinico “P. Giaccone”, University of Palermo, Via Liborio Giuffré 5, 90127 Palermo, Italy; fabris.fv@gmail.com (F.V.); calogera.amato92@gmail.com (C.A.); irene_melfa@yahoo.it (G.M.); giusi_orlando@hotmail.it (G.O.); 4 Unit of General and Oncology Surgery, Department of Surgical Oncological and Oral Sciences, Policlinico “P. Giaccone”, University of Palermo, Via L. Giuffré, 5, 90127 Palermo, Italy; stefania.saverino@hotmail.it (S.S.); antonella.lopes22@gmail.com (A.L.); giuseppa.graceffa@unipa.it (G.G.); 5 Department of Health Promotion Sciences Maternal and Infantile Care, Internal Medicine and Medical Specialties (PROMISE), Section of Endocrinology, University of Palermo, 90127 Palermo, Italy; pierina.richiusa@policlinico.pa.it; 6 Unit of Clinical Epidemiology and Tumor Registry, Department of Laboratory Diagnostics, Policlinico “P. Giaccone”, University of Palermo, Via L. Giuffré, 5, 90127 Palermo, Italy; mazzolasergio3@gmail.com

**Keywords:** thyroid carcinoma, inflammation, systemic immune-inflammation index (SII), neutrophil-to-lymphocyte ratio (NLR), biomarkers, tumor microenvironment

## Abstract

Background: Inflammation within tumor microenvironments has been correlated to numerous malignancies. This study aims to explore its significance in thyroid cancer (TC). Methods: Retrospective analysis of 157 thyroid carcinomas and 40 benign cases involved initial univariate analysis. The value of neutrophils/value of lymphocytes (NLR), value of platelets/value of lymphocytes (PLR), value of lymphocytes/value of monocytes (LMR), and value of platelets × value of neutrophils/value of lymphocytes (SII) indexes were related to TC characteristics and number and location of involved lymph nodes using χ^2^ or Fischer’s exact tests for categorical variables and Student’s *t*-tests for continuous ones. A 1:1 propensity score matching balanced malignant and benign TC groups based on age, sex, and tumor size was used. Post-matching, a multivariate logistic model integrated sex, age, Central lymph node metastases (CLNM), and SII index. Statistically significant immune index values underwent ROC curve analysis for determining cut-offs. Among the 157 malignant TC, median test and density plots were performed. Results: The SII index emerged as a predictor of malignancy in both univariate and multivariate analyses (*p*-value = 0.0202). The ROC curve indicated a cut-off SII value of 465.71, (specificity = 58% [95% CI: 0.43–0.73]; sensitivity = 80% [95% CI: 0.68–0.93]). Median SII index values for tumor sizes of 1 and >1 were 522.8 and 654.8, respectively (*p*-value = 0.016). When central lymph nodes metastases(CLNMs) was considered (CLNM = 0 vs. CLNM > 0), median SII values were 530.7 and 1121.7, respectively (*p*-value = 0.011). Conclusions: The SII index appears to be a valuable tool in the presence of TC, showing correlations with malignancy, tumor size, and CLNMs.

## Simple Summary

Thyroid cancer is a common malignancy, and researchers are exploring new ways to improve its diagnosis. Inflammation plays a key role in cancer development, and this study investigates whether specific blood markers related to the body’s immune response can help detect thyroid cancer and predict its severity. By analyzing blood samples from patients with both benign and malignant thyroid conditions, the researchers identified a marker called the systemic immune-inflammation index as a potential predictor of thyroid cancer. Higher systemic immune-inflammation index values were linked to larger tumors and greater lymph node involvement, suggesting that this marker could help doctors assess cancer progression. These findings highlight the importance of inflammation in thyroid cancer and suggest that blood tests measuring the systemic immune-inflammation index might assist in early diagnosis and treatment planning.

## 1. Introduction

Papillary thyroid carcinoma (PTC) is the most common thyroid malignancy, representing the predominant type among endocrine tumors [1]. Most thyroid carcinomas (TC) exhibit indolent or minimally aggressive behavior. However, secondary spread to cervical lymph nodes (VI, III-IV, V, and II levels) is relatively frequent [2,3]. Lymph node metastasis (LNM) can occur in larger, more aggressive tumors as well as in the classic variant of papillary microcarcinomas [4,5]. Additionally, a minority of these tumors may become refractory to standard treatments such as radioiodine, leading to mortality. These cases might benefit from alternative therapies, such as molecular targeted inhibitors and immunotherapies [6,7,8,9,10,11]. Recent studies, including the article by Pu et al. (2021) [6], have emphasized the role of the tumor ecosystem and immune microenvironment in PTC progression. At the beginning of the 21st century, the link between inflammation and cancer was explored, particularly with reference to Virchow’s hypothesis about leukocytes in neoplastic tissues and the influence of inflammatory cells and cytokines on tumor growth and progression [12]. This growing body of experimental data has informed clinical trials assessing the significance of simple blood immune indexes in patients undergoing thyroidectomy for PTC. Indices such us neutrophil-to-lymphocyte ratio (NLR), platelet-to-lymphocyte ratio (PLR), lymphocyte-to-monocyte ratio (LMR), and systemic immune-inflammation (SII) index have been evaluated, demonstrating their predictive value in assessing PTC risk [13,14].

However, certain aspects remain unclear, such as the threshold values of these indexes for malignancy and their correlation with LNM, including central (CLNM) and lateral (LLNM) compartments [15,16]. It is also uncertain whether some indexes are more effective than others in correlating with tumor characteristics and extent.

This study aims to investigate the clinical significance of preoperative blood immune indexes on TC extent and to establish a predictive clinical model for diagnosis.

## 2. Materials and Methods

A retrospective study was conducted in collaboration between the Department of Surgical, Oncological, and Stomatological Disciplines at the University of Palermo and the Department of Surgery at Sapienza University of Rome. Clinical records of thyroid cancer cases of various histotypes, excluding poorly differentiated and anaplastic forms, were analyzed. To achieve a homogeneous patient sample, we included in the study all patients treated from 1 January 2018 to 31 December 2022. Since the study period included the COVID-19 pandemic, at the time of blood parameters assessment, all patients underwent a COVID-19 PCR test with results being negative for COVID-19. Moreover, all patients did not show clinical signs of other virus infections.

The inclusion criteria were as follows: (1) histological diagnosis of malignant thyroid tumor; (2) age over 18 years; (3) diagnosis of a solitary or dominant nodule (remaining nodules, if present, <1 cm in size); (4) total thyroidectomy with or without central neck dissection, with or without lateral neck dissection; (5) complete clinical data with at least 1-year follow-up. Exclusion criteria included highly aggressive cancers (e.g., anaplastic, poorly differentiated, or insular carcinomas), microcarcinomas, gross invasion of surrounding tissues (T3b or more), patients treated with thyroid lobectomy, concurrent tumors or previous malignancies, and evidence of hematologic, autoimmune, or inflammatory diseases.

Additionally, data from patients with benign nodules (BN) undergoing thyroidectomy were collected. Inclusion criteria for this group were as follows: (1) histological diagnosis of BN; (2) diagnosis of a dominant nodule (remaining nodules < 1 cm in size); (3) total thyroidectomy; (4) complete clinical records. Exclusions mirrored those of the first group, including patients treated with lobectomy and those with similar diseases.

Patients suffering from Hashimoto’s Thyroiditis and diagnosed with thyroid autoantibodies or histopathology were also excluded.

Lobectomies were excluded from both groups to verify total foci number, exclude tumor foci in remnant tissue, and ensure thyroglobulin reliability during follow-up.

This study was conducted in accordance with the guidelines of the Declaration of Helsinki of 1964 and its later amendments. All patients provided their informed consent for the surgical procedure and consented to the processing of their personal data. The clinical data recorded in the database (XLSX) were handled with full respect for anonymity.

### 2.1. Variables

Variables included demographic data (age, sex); nodule size (simplified TNM criteria [12]); cancer histotype and variant; number of foci; lymph nodes removed (central and/or lateral); postoperative persistence/recurrence (PR); and blood immune indexes (NLR, PLR, LMR, SII) measured pre-thyroidectomy.

### 2.2. Surgical Technique

The interventions were performed after obtaining informed consent from the patients. They followed established guidelines, including selective ligation or division of thyroid artery branches and the use of energy devices. Total thyroidectomy was performed when nodules were in the contralateral lobe. Nerve monitoring assisted all the procedures. Hemostatic agents were applied before closure.

### 2.3. Follow-Up

Thyroglobulin was the marker for differentiated TC (DTC) follow-up [17]. For medullary thyroid carcinoma (MTC), Calcitonin levels were measured using chemiluminescence assay. Remission was indicated by undetectable or normal range Calcitonin levels [18].

### 2.4. Statistical Analysis

Initial univariate analysis compared malignant lesions with benign nodules using Fisher’s exact test, Chi-square test, and Student’s *t*-test. A 1:1 matching analysis was carried out using the propensity score method to balance case and control groups based on age, sex, and nodule size. The average distances of the variables between the two groups were below the value of 0.2. Based on the stepwise model, variables included in the multivariate logistic modeling were sex, age, CLNM, and SII index. ROC curves were generated for significant variables to determine sensitivity, specificity, and cut-off values. Finally, median tests in the malignancy group assessed SII index associations with tumor size, variants, foci, lymph nodes, and postoperative Thyroglobulin. Density plots visualized SII index distributions.

The analysis was conducted using RStudio v3.4.1 and R software v2.1, with specificity and sensitivity calculated using the ‘pROC’ package.

## 3. Results

This study included a total of 197 patients. Of these, 157 patients with thyroid carcinoma and 40 patients with single or predominant benign nodular lesions were recruited.

Univariate analysis (Table 1) revealed that the two groups were heterogeneous in nodule size; however, in terms of age and sex, the groups appeared balanced. The NLR (with a *p*-value = 0.0506) and the SII index (with a *p*-value < 0.01) were statistically significant.

In the multivariate logistic model (Table 2), the covariates included, based on the stepwise model, sex, age, CLNM, and SII index. The SII index remained statistically significant in the multivariate analysis as well.

**Figure 1:**
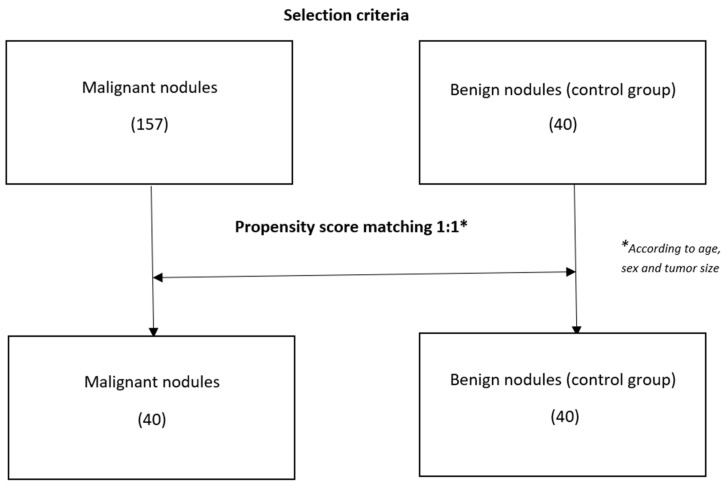
After the matching, we obtained two groups of equal size, balanced in terms of age, sex, and tumor size.

The 1:1 matching (Figure 1) was carried out using the propensity score method, based on covariates of age, sex, and tumor size, identified a homogeneous group of TC for comparison in the logistic regression model applied to the benign nodule group. Logistic multivariate analysis shows that only the SII index is statistically significant as a predictor of tumor malignancy with an OR = 1.002 and a *p*-value 0.0202.

**Figure 2:**
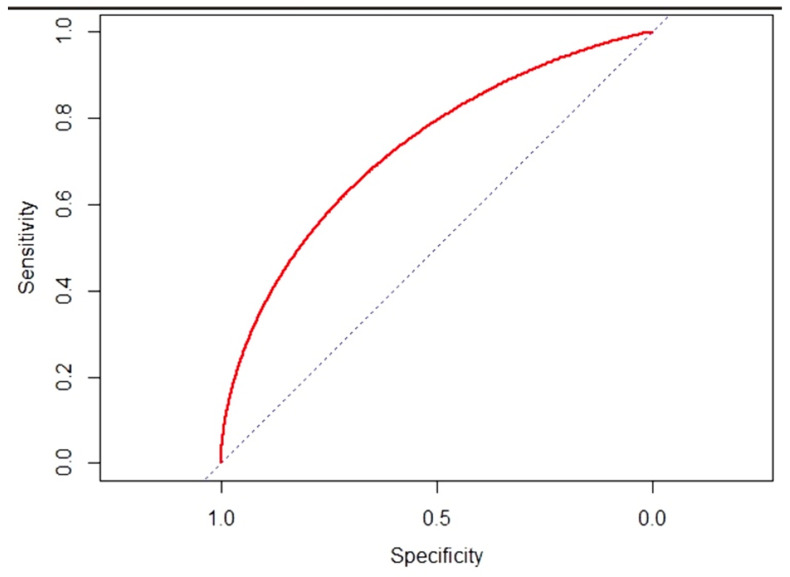
ROC curve—SII index. As evidenced by the ROC curve, the SII index proved to be a good predictor of malignancy.

In the group of 157 malignancy cases, the median test was applied in univariate analysis to verify whether the SII index value increased based on the following variables: tumor size (categorized as 1 vs. >1); variants (categorized as 1, 2–5, 6); FOCI (categorized as 1 and 2–3); CLNM (categorized as 0 vs. >0); LLNM (categorized as 0 vs. >0); PR (categorized as 0 and 1). The ROC curve calculated on the SII index (Figure 2) showed that this indicator is predictive of malignancy with a threshold value of 465.71, specificity = 0.58 with 95% CI (0.43–0.73) and sensitivity = 0.80 with 95% CI (0.68–0.93) and an AUC = 0.7292.

Comparing SII index values based on tumor size (1 vs. >1), the median SII index value for a tumor size of 1 is Me = 522.8, while the median SII index value for tumor sizes >1 is Me = 654.8. These two values are statistically significant (*p*-value = 0.016).

**Figure 3:**
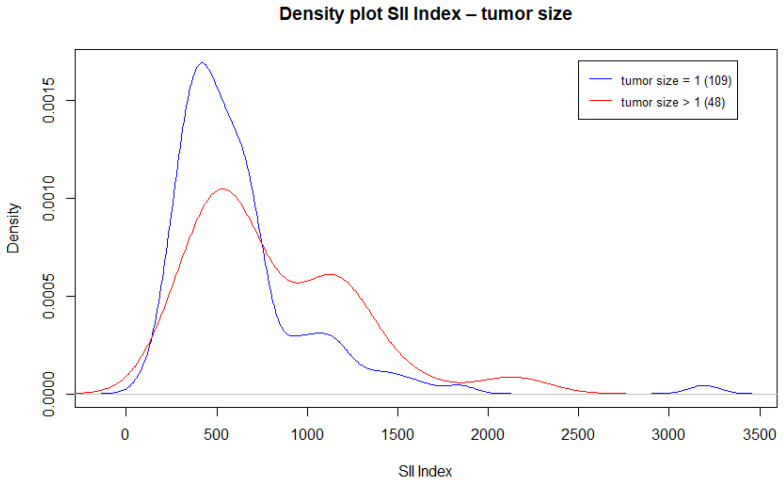
Density plot SII index—tumor size. In malignant tumors larger than T1, the SII index tends to distribute significantly around values that range between 500 and 1000.

The density plot (Figure 3) highlights that the curve for SII index values for tumor size 1 shows the highest percentage of patients clustered around the SII index value of 500 (above the cut-off 465.71), while the curve for SII index values for tumor sizes >1 is shifted towards higher SII index values, showing a higher percentage of patients with values above 1000 compared to tumor size 1.

Comparing SII index values based on the variant variable (1 vs. 2–5), the median SII index value for variant 1 is Me = 544.0, while the median SII index value for variant 2–5 is Me = 576.2. These two values are not statistically significant (*p*-value = 0.607).

Comparing SII index values based on the FOCI variable (1 vs. 2–3), the median SII index value for FOCI 1 is Me = 582.2, while the median SII index value for FOCI 2–3 is Me = 530.3. These two values are not statistically significant (*p*-value = 0.575).

Comparing SII index values based on CLNM (0 vs. >1), the median SII index value for CLNM 0 is Me = 530.7, while the median SII index value for CLNM >1 is Me = 1121.7. These two values are statistically significant (*p*-value = 0.011).

**Figure 4:**
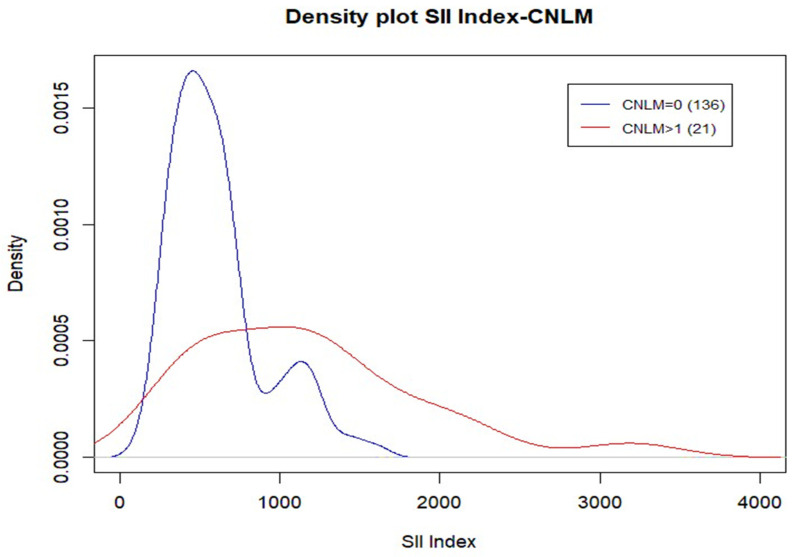
Density plot SII index—CLNM. Despite the wider distribution of the SII index in patients presenting with level VI metastasis, it is evident that the peak concentration is around 1000, in contrast to patients without central metastases, where the peak is much lower.

The density plot (Figure 4) highlights that the curve for SII index values for CLNM 1 shows the highest percentage of patients clustered around the SII index value of 500 (above the cut-off 465.71), while the curve for SII index values for CLNM >1 is shifted towards higher SII index values, showing a higher percentage of patients with values equal to or above 1000.

Comparing SII index values based on LLNM (0 vs. >1), the median SII index value for LLNM 0 is Me = 546.6, while the median SII index value for LLNM >1 is Me = 1135.2. These two values are statistically significant (*p*-value = 0.0467). It should be noted that the lack of significance is due to the fact that there are 144 patients with LLNM 0 and only 13 with LLNM >1.

Comparing SII index values based on PR (0 vs. 1), the median SII index value for PR 0 is Me = 548.2; while the median SII index value for PR 1 is Me = 1148.4. These two values are not statistically significant (*p*-value = 0.1160). It should be noted that the lack of significance is due to the fact that there are 151 patients with PR 0 and only 6 with PR 1.

## 4. Discussion

Possible interrelationships between the inflammatory cascade and cancer have been hypothesized since the 19th century and reexamined at the beginning of the 21st century [12,13,14].

Tumor progression has been linked to transcription factors, cytokines, chemokines, and infiltrating leukocytes. Chronic inflammation, in fact, plays a role in cancer proliferation, survival, angiogenesis, metastasis, and immune modulation. Notably, inflammatory cytokines play a critical role in promoting genetic instability in cancer cells [19].

The immune microenvironment within tumors plays a pivotal role in both the advancement and the prognosis of various cancers, and this has been studied extensively, particularly in breast cancer. Within this environment, neutrophils are notably prevalent among the immune cells [20], promoting tumor growth and facilitating the spread of breast cancer [21].

Conversely, tumor-infiltrating lymphocytes exhibit anticancer properties, often associated with less advanced cancer stages and considered a positive prognostic factor [22].

A low genetic NLR in tumors has been correlated with a favorable tumor immune microenvironment and improved survival in triple-negative breast cancer [23].

As highlighted in breast cancer, thymic stromal lymphopoietin (TSLP) plays a significant role in the tumor microenvironment, influencing the expansion of CD14+CD16+ monocytes, a subset of immune cells that are involved in inflammation and tissue repair, and modulating cellular metabolism [24].

Immuno-inflammatory markers, assessed both pre- and post-operatively, show additional correlations with prognosis in urothelial, esophageal, and pancreatic cancers [25,26,27,28].

Recent studies have spotlighted inflammation’s role in the tumor microenvironment of TC, especially PTC. Patients with DTC, particularly those with multifocality, exhibit higher SII values than the healthy population, suggesting inflammation’s potential role in these malignancies [29]. Single-cell transcriptome sequencing of PTC tissue has revealed a complex ecosystem of cell types, highlighting the intricate interaction between thyroid cancer cells and the immune microenvironment in PTC [30]. A study on the association between inflammatory biomarkers and lymph node involvement in PTC has identified an independent correlation between SII values and CLNM, suggesting that inflammatory markers are significant predictors of tumor progression in TC [31]. Tumor diameter and preoperative SII are also independent risk factors for LLNM in PTC patients, reinforcing the predictive value of inflammatory markers for tumor progression and emphasizing the significance of blood immune indexes in understanding PTC biology [16]. A preoperative prediction model for PTC, incorporating ultrasound characteristics, thyroid-stimulating hormone (TSH), and inflammatory markers, has demonstrated that inflammation-related markers can enhance the preoperative diagnostic process [32]. High NLR are also correlated with larger tumor sizes and extrathyroidal extension in PTC [10]. Finally, inflammatory biomarkers like NLR, LMR, and PLR may be effective tools in predicting malignancy in indeterminate thyroid nodules [33].

Recently, the importance of the tumor microenvironment has also been investigated in cancers associated with the RET oncogene. Mulligan et al. [34] highlighted how RET signaling, driven by both oncogenic mutations and tumor microenvironment interactions, promotes extracellular matrix remodeling, cell migration, and perineural invasion, processes closely linked to the progression and metastasis of MTC. Targeting RET pathways may therefore provide a therapeutic strategy not only for malignancy but also for modulating the tumor microenvironment.

Our study confirms the significant role of inflammation and immune responses in PTC progression and metastasis. The integration of immunological markers with clinical and histological data is relatively underexplored in thyroid cancer research, offering new insights into disease progression and providing opportunities for diagnostic improvement.

As demonstrated elsewhere, lymphocytic infiltrate in thyroid tissue may act as a defense mechanism, potentially triggered by aberrantly glycosylated thyroglobulin in the colloid of follicles with early dysplastic changes [35,36]. This suggests a broader context in which immune markers, including the presence of lymphocytic infiltrate, might help elucidate the immune landscape in thyroid disease progression and carcinogenesis.

Preoperative SII values appear crucial for predicting malignancy, enhancing diagnostic accuracy and aiding clinicians in making better-informed decisions, especially in borderline cases.

The SII demonstrates good predictive ability even in patients without lymph node metastasis. This is particularly relevant for nodules smaller than 2 cm without lymph node involvement, where the need for surgical intervention remains debated.

Patients with low-risk papillary thyroid microcarcinoma who undergo thyroidectomy may experience significant decision regret, especially in the case of small tumors [37]. In this context, the integration of SII as a predictive biomarker may have important clinical value, helping to guide more tailored treatment strategies and reducing unnecessary surgeries, with psychological benefits as well.

Moreover, the ability to stratify patients based on LNM risk and tumor aggressiveness using SII values can lead to more personalized treatment plans. This stratification can help determine the extent of surgery and the intensity of follow-up required. Patients with suspected higher risks of aggressive disease or LNM could be planned for more extended surgical interventions, especially if these biomarkers are associated with further prognostic data [38,39].

The use of SII as a prognostic marker could also improve postoperative follow-up strategies.

Our study confirms the role of immune function, inflammation, and tumor microenvironment in the prognosis of thyroid cancer. Understanding the individual patient’s disease profile can lead to more tailored therapeutic strategies.

Our study presents several notable aspects: first, it evaluates the predictive value of blood immune indexes across all thyroid tumors examined, rather than focusing solely on papillary histotype. This approach is naturally limited by the low frequency of non-papillary histotypes, which prevented separate analysis of follicular carcinomas and precluded statistical evaluations for medullary carcinomas due to the small sample size. Nonetheless, despite the limitation of grouping heterogeneous forms (different PTC variants, follicular carcinomas, and NIFTP-UMP), the similarity in SII index between these (SII = 576.2) and the classic PTC variant (SII = 544) may suggest that SII index is not strongly influenced by tumor histotype, although this hypothesis requires validation in larger cohorts. Therefore, for now, we can consider the consolidated results on our sample as applicable to TC regardless of histotype or variant.

Another important step is the identification of a threshold value of the SII (465.71) predictive of malignancy. These data could serve as additional criteria for guiding clinical decisions in indeterminate nodules. Its utility appears greater for larger lesions, as shown by the density plot, which displays a concentration of SII values around 1000 for tumors > 2 cm.

Of particular interest, due to its potential therapeutic implications, is the difference (clearly visualized in the corresponding density plot) between tumors with negative CLNMs (SII ~500) and those positive CLNMs (SII ~1000). Although statistical significance was not reached (likely due to the small sample size with positive LLNMs), a marked difference was also observed between patients with negative LLNMs and positive LLNMs. It should be noted that LLNM positive cases almost always presented with concurrent metastases at level VI.

Beyond the limitations already mentioned regarding specific variables, a key limitation lies in the size difference we observed between the group of patients with TC and the group of patients with benign nodules. This is due to the lack of surgical indications for small nodules without a strong clinical, instrumental, and cytological suspicion. For the same reason, many of the patients with malignant thyroid lesions, and all of those with benign nodules, had a ‘dominant’ but not exactly ‘solitary’ nodule. For secondary nodules, we applied a 1 cm size cutoff.

Moreover, the limited sample size of this study should be considered along with the lack of external validation, meaning the absence of validation of our predictive factors in independent cohorts or settings.

Furthermore, recent advances in diagnostic methods have significantly reduced unnecessary thyroidectomies, an improvement that nonetheless influenced the design of our study.

Despite its methodological limits, our study crucially expands the understanding of blood immune indexes in thyroid cancer, suggesting their broad applicability across various histotypes and highlighting their potential as novel diagnostic and prognostic tools.

This contributes significantly to refining patient management and advancing personalized treatment approaches.

## 5. Conclusions

This study highlights the role of inflammation in the natural history of thyroid carcinoma. The SII index, alongside theNLR, emerges as a key marker in TC. Our findings suggest that SII values are closely linked to malignancy, tumor size, and lymph node metastasis. Further research is needed to explore potential differences among various histotypes and variants of thyroid tumors.

These results support the evaluation of the SII index as an additional diagnostic tool for thyroid nodules and suspected lymph node metastasis. The measurement of these indicators is extremely straightforward, as it is based on basic laboratory tests; consequently, their cost is negligible. Moreover, they do not cause any delays in diagnostic pathways. All things considered, if further investigations confirm their association with thyroid carcinomas, these markers could be routinely integrated into thyroid nodule evaluation protocols, given their excellent cost effectiveness.

## Figures and Tables

**Table 1:** Univariate analysis.

UNIVARIATE ANALYSIS				
Variable	Benign (40)	Malignant (157)	Total 197	*p*-Value
AGE median (range)	50 (35–78)	54 (24–87)	52 (24–87)	0.684
SEX n (%)				
Male (M)	9 (4%)	49 (25%)	58 (29%)	0.334
Female (F)	31 (16%)	108 (55%)	139 (71%)	
NODULE SIZE * n (%)				
1	3 (2%)	109 (55%)	112 (57%)	<0.001
2	27 (13%)	31 (16%)	58 (29%)	
3	10 (5%)	15 (8%)	25 (13%)	
4	0 (0%)	2 (1%)	2 (1%)	
N FOCI n (%)				
1	40 (20%)	119 (61%)	159 (81%)	<0.001
2	0	6 (3%)	6 (3%)	
3	0	32 (16%)	32 (16%)	
CLNM n (%)				
0	40 (100%)	136 (87%)	176 (81%)	0.041
1–11	0	21 (13%)	21 (19)	
LLNM n (%)				
0	40 (100%)	144 (92%)	184 (93%)	0.060
1–7	0	13 (8%)	13 (7%)	
RECURRENCE n (%)				
0	40 (100%)	151 (96%)	191 (97%)	0.209
1	0	6 (4%)	6 (3%)	
INDEXES median (range)				
NLR	2.08 (0.82–3.96)	2.48 (0.03–13.15)		0.0506
LMR median (range)	4.18 (2.21–8.05)	3.95 (1.33–8.23)		0.362
PLR median (range)	124.57 (63.21–267.02)	139.90 (1.24–400.00)		0.181
SII index median (range)	495.37 (164.34–1072.71)	684.53 (119.85–3194.85)		<0.001

A univariate analysis was carried out to assess differences between groups using the Student’s *t*-test. * Nodule size was categorized based on simplified TNM criteria. CLNM: central lymph node metastases; LLNM: lateral lymph node metastases; NLR: neutrophil-to-lymphocyte ratio; LMR: lymphocyte-to-monocyte ratio; PLR: platelet-to-lymphocyte ratio; SII: systemic immune inflammation.

**Table 2:** Multivariate logistic model.

MULTIVARIATE ANALYSIS				
VARIABLE	OR	CI (Inf) 95%	CI (Sup) 95%	*p*-Value
Sex (M)	0.000020	4.2 × 10^−10^	3.31 × 10^−1^	0.0736
Age	0.9897	9.48 × 10^−1^	1.032	0.0669
CLNM	6.8 × 10^7^	1.84 × 10^−28^	1.27 × 10^243^	0.9918
SII_Index	1.002	1.0004	1.004	0.0202
Sex:Age	1.1662	1.03	1.46	0.0599

Variables were included in the analysis based on stepwise model. CI: confidence interval; M: male; CLNM: central lymph node metastases; SII: systemic immune inflammation.

## Data Availability

Data are not available due to privacy and ethics restrictions.

## References

[1] Houten R., Fleeman N., Kotas E., Boland A., Lambe T., Duarte R. (2021). A systematic review of health state utility values for thyroid cancer. Qual. Life Res..

[2] Dolidze D.D., Shabunin A.V., Mumladze R.B., Vardanyan A.V., Covantsev S.D., Shulutko A.M., Semikov V.I., Isaev K.M., Kazaryan A.M. (2022). A Narrative Review of Preventive Central Lymph Node Dissection in Patients with Papillary Thyroid Cancer—A Necessity or an Excess. Front. Oncol..

[3] Censi S., Galuppini F., Clausi C., Battheu F., Manso J., Piva I., Corvaglia S., Pedron M.C., Mondin A., Iacobone M. (2024). Tumor Grade and Molecular Characteristics Associated with Survival in Sporadic Medullary Thyroid Carcinoma. Thyroid.

[4] Yan L., Blanco J., Reddy V., Al-Khudari S., Tajudeen B., Gattuso P. (2019). Clinicopathological features of papillary thyroid microcarcinoma with a diameter less than or equal to 5 mm. Am. J. Otolaryngol..

[5] Luo Y., Zhao Y., Chen K., Shen J., Shi J., Lu S., Lei J., Li Z., Luo D. (2019). Clinical analysis of cervical lymph node metastasis risk factors in patients with papillary thyroid microcarcinoma. J. Endocrinol. Investig..

[6] Pu W., Shi X., Yu P., Zhang M., Liu Z., Tan L., Han P., Wang Y., Ji D., Gan H. (2021). Single-cell transcriptomic analysis of the tumor ecosystems underlying initiation and progression of papillary thyroid carcinoma. Nat. Commun..

[7] Baldini E., Presutti D., Favoriti P., Santini S., Papoff G., Tuccilli C., Carletti R., Di Gioia C., Lori E., Ferent I.C. (2022). In Vitro and In Vivo Effects of the Urokinase Plasminogen Activator Inhibitor WX-340 on Anaplastic Thyroid Cancer Cell Lines. Int. J. Mol. Sci..

[8] Tiedje V., Fagin J.A. (2020). Therapeutic breakthroughs for metastatic thyroid cancer. Nat. Rev. Endocrinol..

[9] Mehnert J.M., Varga A., Brose M.S., Aggarwal R.R., Lin C.C., Prawira A., de Braud F., Tamura K., Doi T., Piha-Paul S.A. (2019). Safety and antitumor activity of the anti-PD-1 antibody pembrolizumab in patients with advanced, PD-L1-positive papillary or follicular thyroid cancer. BMC Cancer.

[10] Papale F., Cafiero G., Grimaldi A., Marino G., Rosso F., Mian C., Barollo S., Pennelli G., Sorrenti S., De Antoni E. (2013). Galectin-3 expression in thyroid fine needle cytology (t-FNAC) uncertain cases: Validation of molecular markers and technology innovation. J. Cell Physiol..

[11] Gabillard J.C., Ulisse S., Baldini E., Sorrenti S., Cremet J.Y., Coccaro C., Prigent C., D’Armiento M., Arlot-Bonnemains Y. (2011). Aurora-C interacts with and phosphorylates the transforming acidic coiled-coil 1 protein. Biochem. Biophys. Res. Commun..

[12] Balkwill F., Mantovani A. (2001). Inflammation and cancer: Back to Wirchow?. Lancet.

[13] Ceylan Y., Kumanlioglu K., Oral A., Ertan Y., Ozcan Z. (2019). The correlation of clinicopathological findings and neutrophil-to-Lymphocyte and platelet-to Lymphocyte ratios in papillary thyroid carcinoma. Mol. Imaging Radionucl. Ther..

[14] Mantovani A., Allavena P., Sica A., Balkwill F. (2008). Cancer-related inflammation. Nature.

[15] Tuttle R.M., Haugen B., Perrier N.D. (2017). Updated American Joint Committee on Cancer/Tumor-Node-Metastasis Staging System for Differentiated and Anaplastic Thyroid Cancer (Eighth Edition): What Changed and Why?. Thyroid.

[16] Zhao L., Zhou T., Zhang W., Wu F., Jiang K., Lin B., Zhan S., Hu T., Tang T., Zhang Y. (2022). Blood immune indexes can predict lateral lymph node metastasis of thyroid papillary carcinoma. Front. Endocrinol..

[17] Giovanella L. (2020). Circulating biomarkers for the detection of tumor recurrence in the postsurgical follow-up of differentiated thyroid carcinoma. Curr. Opin. Oncol..

[18] Kloos R.T., Eng C., Evans D.B., Francis G.L., Gagel R.F., Gharib H., Moley J.F., Pacini F., Ringel M.D., American Thyroid Association Guidelines Task Force (2009). Medullary thyroid cancer: Management guidelines of the American Thyroid Association. Thyroid.

[19] Colotta F., Allavena P., Sica A., Garlanda C., Mantovani A. (2009). Cancer-related inflammation, the seventh hallmark of cancer: Links to genetic instability. Carcinogenesis.

[20] Hajizadeh F., Aghebati Maleki L., Alexander M., Mikhailova M.V., Masjedi A., Ahmadpour M., Hashemi V., Jadidi-Niaragh F. (2021). Tumor-associated neutrophils as new players in immunosuppressive process of the tumor microenvironment in breast cancer. Life Sci..

[21] Gregory A.D., Houghton A.M. (2011). Tumor-associated neutrophils: New targets for cancer therapy. Cancer Res..

[22] Dieci M.V., Miglietta F., Guarneri V. (2021). Immune infiltrates in breast cancer: Recent updates and clinical implications. Cells.

[23] Tokumaru Y., Oshi M., Murthy V., Tian W., Yan L., Angarita F.A., Nagahashi M., Matsuhashi N., Futamura M., Yoshida K. (2021). Low intratumoral genetic neutrophil-to-lymphocyte ratio (NLR) is associated with favorable tumor immune microenvironment and with survival in triple negative breast cancer (TNBC). Am. J. Cancer Res..

[24] Marcella S., Braile M., Grimaldi A.M., Soricelli A., Smaldone G. (2025). Exploring thymic stromal lymphopoietin in the breast cancer microenvironment: A preliminary study. Oncol. Lett..

[25] Jan H.C., Wu K.Y., Tai T.Y., Weng H.Y., Yang W.H., Ou C.H., Hu C.Y. (2022). The Systemic Immune-Inflammation Index (SII) Increases the Prognostic Significance of Lymphovascular Invasion in Upper Tract Urothelial Carcinoma After Radical Nephroureterectomy. Cancer Manag. Res..

[26] Xu X., Jing J. (2022). Inflammation-related parameter serve as prognostic biomarker in esophageal squamous cell carcinoma. Front. Oncol..

[27] De Pasquale L., Lori E., Bulfamante A.M., Felisati G., Castellani L., Saibene A.M. (2021). Evaluation of Wisconsin and CaPTHUS Indices Usefulness for Predicting Monoglandular and Multiglandular Disease in Patients with Primary Hyperparathyroidism through the Analysis of a Single-Center Experience. Int. J. Endocrinol..

[28] Schlanger D., Popa C., Pașca S., Seicean A., Al Hajjar N. (2022). The role of systemic immuno-inflammatory factors in resectable pancreatic adenocarcinoma: A cohort retrospective study. World J. Surg. Oncol..

[29] Kars A., Sahin A., Kılıc K., Sakat M.S., Bilen A. (2022). Systemic immune inflammation index in differentiated thyroid cancers. Acta Otorhinolaryngol. Ital..

[30] Yan T., Qiu W., Weng H., Fan Y., Zhou G., Yang Z. (2021). Single-Cell Transcriptomic Analysis of Ecosystems in Papillary Thyroid Carcinoma Progression. Front. Endocrinol..

[31] Zhang Z., Xia F., Wang W., Huang Y., Li X. (2021). The systemic immune-inflammation index-based model is an effective biomarker on predicting central lymph node metastasis in clinically nodal-negative papillary thyroid carcinoma. Gland. Surg..

[32] Tang Z.W., Li X.X., Luo J. (2023). Development and validation of the nomogram based on ultrasound, thyroid stimulating hormone, and inflammatory marker in papillary thyroid carcinoma: A case-control study. Transl. Cancer Res..

[33] Gambardella C., Mongardini F.M., Paolicelli M., Bentivoglio D., Cozzolino G., Ruggiero R., Pizza A., Tolone S., Del Genio G., Parisi S. (2023). Role of Inflammatory Biomarkers (NLR, LMR, PLR) in the Prognostication of Malignancy in Indeterminate Thyroid Nodules. Int. J. Mol. Sci..

[34] Mulligan L.M. (2019). GDNF and the RET Receptor in Cancer: New Insights and Therapeutic Potential. Front. Physiol..

[35] Cabibi D., Giannone A.G., Bellavia S., Lo Coco R., Lo Bianco A., Formisano E., Scerrino G., Graceffa G. (2023). Serum Anti-Thyroglobulin Autoantibodies Are Specific in Predicting the Presence of Papillary-like Nuclear Features and Lymphocytic Infiltrate in the Thyroid Gland. Diagnostics.

[36] Bellini M.I., Lori E., Forte F., Lauro A., Tripodi D., Amabile M.I., Cantisani V., Varanese M., Ferent I.C., Baldini E. (2022). Thyroid and renal cancers: A bidirectional association. Front. Oncol..

[37] Li G., Li R., Zhong J., Chen W., Shuai J., Chen M., Deng F., Wei T., Tang H., Li Z. (2025). A multicenter cohort study of thyroidectomy-related decision regret in patients with low-risk papillary thyroid microcarcinoma. Nat. Commun..

[38] Graceffa G., Orlando G., Cocorullo G., Mazzola S., Vitale I., Proclamà M.P., Amato C., Saputo F., Rollo E.M., Corigliano A. (2021). Predictors of Central Compartment Involvement in Patients with Positive Lateral Cervical Lymph Nodes According to Clinical and/or Ultrasound Evaluation. J. Clin. Med..

[39] Wu X., Li B., Zheng C., He X. (2023). Risk factors for skip metastasis in patients with papillary thyroid microcarcinoma. Cancer Med..

